# Orders of Healthier Adult Menu Items in a Full-Service Restaurant Chain with a Healthier Children’s Menu

**DOI:** 10.3390/nu12113253

**Published:** 2020-10-23

**Authors:** Megan P. Mueller, Eleanor T. Shonkoff, Sara C. Folta, Stephanie Anzman-Frasca, Christina D. Economos

**Affiliations:** 1 Department of Food Science and Human Nutrition and Colorado School of Public Health, Colorado State University, Fort Collins, CO 80523, USA; 2 School of Health Sciences, Merrimack College, North Andover, MA 01845, USA; shonkoffe@merrimack.edu; 3 Friedman School of Nutrition Science and Policy, Tufts University, Boston, MA 02111, USA; Sara.Folta@tufts.edu (S.C.F.); christina.economos@tufts.edu (C.D.E.); 4 Department of Pediatrics, Jacobs School of Medicine and Biomedical Sciences, University at Buffalo, Buffalo, NY 14214, USA; safrasca@buffalo.edu; 5 Center for Ingestive Behavior Research, University at Buffalo, Buffalo, NY 14260, USA; 6 ChildObesity180, Tufts University, Boston, MA 02111, USA

**Keywords:** healthy menu changes, adult menu orders, healthier items

## Abstract

This study evaluated orders of adult menu items designated as healthier at the Silver Diner, a regional full-service restaurant chain serving over 4 million customers annually. This restaurant implemented a healthier children’s menu in April 2012. Orders of adult menu items were abstracted from before (September 2011–March 2012; PRE; *n* = 1,801,647) and after (September 2012–March 2013; POST; *n* = 1,793,582) the healthier children’s menu was introduced. Entrées, appetizers, and sides listed as healthier options on the menu were coded as healthier. PRE to POST changes in the percentage of orders of healthier items, soda, and dessert were evaluated using McNemar tests of paired proportions. Orders of healthier entrées, appetizers, and sides on the adult menu increased PRE to POST (8.9% to 10.4%, 25.5% to 27.5%, and 7.3% to 9.3%, respectively), and soda and dessert orders decreased (23.2% to 21.7% and 29.0% to 28.3%, respectively). All shifts were statistically significant (*p* < 0.0001). Our findings demonstrate improvements in orders of healthier adult menu options during the same time frame as a healthy children’s menu change. Future research can help elucidate mechanisms to inform future health promotion efforts in restaurants in ways that have the potential to impact both adults and children.

## 1. Introduction

Restaurants continue to be an increasing source of food in the American diet and are a significant source of excess calories, saturated fat, sugar, and sodium [1,2,3]. Restaurants stand to make a significant impact on public health by encouraging the consumption of healthy foods.

In April 2012, the Silver Diner, a restaurant chain serving over 4 million people annually in the Mid-Atlantic region of the United States, introduced a healthier children’s menu. This new menu included more entrées that met Kids LiveWell (Washington, DC, USA) nutrition criteria [4], all meals automatically bundled with fruit and non-fried vegetable sides, and the removal of French Fries and fountain beverages (referred to herein as soda). Previously, we examined children’s menu orders before (September 2011–March 2012; PRE) to after (September 2012–March 2013; POST) these healthy children’s menu changes. This study revealed significant, lasting increases in the healthfulness of children’s menu orders after the healthy children’s menu changes which persisted at 2 year follow up [5,6,7]. More specifically, orders of nutrient-poor items such as French fries and soda decreased (from 57.0 to 22.0% and from 34.7 to 29.7%, respectively); orders of fruit and vegetable sides, milk, and 100% fruit juices increased (from 29.1 to 63.0%, from 5.5 to 6.9%, from 37.0 to 39.5%, and from 28.3 to 30.8%, respectively) [5].

Healthy menu change interventions in other full service and fast food restaurants have also been associated with healthier orders for children [8,9,10]. The evidence for interventions targeting the adult menu has been mixed [11]; and only menu interventions that used both point of purchase information (i.e., heart healthy symbols or calorie labeling) and increases in the availability of healthier options were consistently associated with increases in orders of healthier items [11]. To our knowledge, there is no evidence evaluating whether orders of adult menu items are healthier when the healthy menu intervention is targeted to the children’s menu. Yet adults can serve as role models for healthier choices in restaurants [12], can help establish norms around healthy dietary practices [13,14,15,16], and engage in other food-related parenting practices that can promote healthier choices in restaurants for children [16,17]. Restaurant meals are also associated with poorer overall dietary quality and chronic disease risk in adults [2,18,19,20]. As such, it is important to understand what adults are ordering in restaurants.

In this secondary analysis, we examined whether changes in orders of standard menu items (referred to herein as the adult menu) also emerged in the context of a healthier children’s menu. We posit that any changes observed in adult orders would not be the result of the adult menu, as there were few changes to the adult menu and no campaigns promoting healthier adult menu options. Moreover, there was a consistent subset of adult menu items designated as healthier selections both before and after the introduction of the healthier children’s menu. We consider potential mechanisms explaining our findings including a temporal shift in the types of customers patronizing the restaurant chain, shifts in cultural norms about healthier restaurant orders, or a spillover effect from the healthier children’s menu that was in place at the restaurant during this same time period. We discuss the implications of this hypothesis-generating work for future research.

## 2. Materials and Methods

### 2.1. Study Design

This repeated cross-sectional study follows previous research evaluating child meal orders after the implementation of a healthier children’s menu [5,6,7]. We conducted a secondary analysis of adult menu order data from 13 outlets of the Silver Diner, a full-service restaurant chain located in the mid-Atlantic region of the US that serves >4 million customers annually. The restaurant introduced the new, healthier children’s menu in April 2012. Additional information about the restaurant and the children’s menu changes have been published elsewhere [5,6,7]. Briefly, this new menu included more entrées that met Kids LiveWell nutrition criteria [1], all meals automatically bundled with fruit and non-fried vegetable sides, and the removal of French Fries and soda. Children’s menus were generally only brought to the table if the dining group included a child or by request.

Aggregated data on orders of adult menu items, which included all items ordered that were not listed on the children’s menu, were abstracted from the restaurant’s central database before (September 2011–March 2012; PRE) and after (September 2012–March 2013; POST) the implementation of the new healthier children’s menu. The time periods corresponded to the same time periods evaluated in previously published work focused on orders of children’s menu items [5,6,7]. A total of 3,595,229 adult entrées were ordered representing 18.8% of all items sold. Other items sold in the restaurant included children’s entrées (1.8% of all items sold), children’s sides (1.0% of all items sold), children’s beverages (1.2% of all items sold), appetizers (0.8% of all items sold), adult menu beverages (14.3% of all items sold), adult sides (23.0% of all items sold), and adult desserts (5.4% of all items sold). Order data were coded in Excel as described below.

We were specifically interested in consumer responses to items designated healthier options on the menu, since previous experimental evidence suggests that consumers tend to view these items as being healthier than items without such distinctions [21]. Healthier adult menu items (entrees, sides, and appetizers—no desserts) were identified based on being (a) listed under the “Healthier Options for Today’s Lifestyles” menu section and denoted with a heart symbol; (b) denoted with a heart symbol in other menu sections to indicate the item is “lower in fat and cholesterol” and/or a “healthier item”; (c) listed as a “healthier choice” side item substitution (i.e., strawberries and egg whites, cholesterol-free egg replacement); and/or (d) seasonal or off-menu selections identified by the restaurant partner as “healthier” using the same heart symbol or as a meal under 600 calories. We collectively refer to these items as healthier options herein. The majority of entrées listed as healthier options (>80%) met cut points for calorie, total fat, cholesterol, and saturated fat needs/limits for a single meal using one-third of the average daily needs/limits for sedentary adults ages 18 and over (Table 1) [22].

The adult menus at PRE and POST had no differences in the percentage of entrées, sides, and appetizers listed as healthier options (Table 2).

In addition to evaluating food items, we examined healthier beverage orders by coding beverages as soda or non-soda, given that soda intake is associated with weight gain, metabolic syndrome, dental caries, and type II diabetes in children and adults [23,24,25]. Soda orders included fountain beverages, fruit drinks, and lemonade, which was consistent with how these orders were coded in previously published research [5,6,7,26]. We also evaluated the most popular beverages ordered overall and indicated which of these beverages were non-soda beverages (Table 3). Only non-alcoholic beverages were included in these analyses to facilitate comparisons with previously published work [5,6,7]. Alcoholic beverages made up only ~5% of beverage orders at both time points.

### 2.2. Analysis

Analyses were conducted in STATA v14 (StataCorp LLC, College Station, TX, USA) and Excel. Descriptive statistics and McNemar tests of paired proportions were used to examine adult menu orders before versus after the healthier children’s menu change. To identify the change in the relative number of orders that included healthier options, we calculated the percentages of items designated as healthier options using the total number of orders that included healthier options divided by the total number of orders overall for each item category (entrées, sides, and appetizers) and at each time point. We then evaluated differences in the percentage of entrée, side, and appetizer orders that included items designated as healthier options from PRE to POST. To be consistent with our previously published work, we also evaluated differences in the percentage of beverage orders that included sodas and the percentage of dessert orders relative to entrée orders during that same time frame. To further inform our discussion of overall trends, we compared differences in the most popular items ordered overall and the most popular items ordered that were designated as healthier options at PRE and POST.

## 3. Results

**Figure 1:**
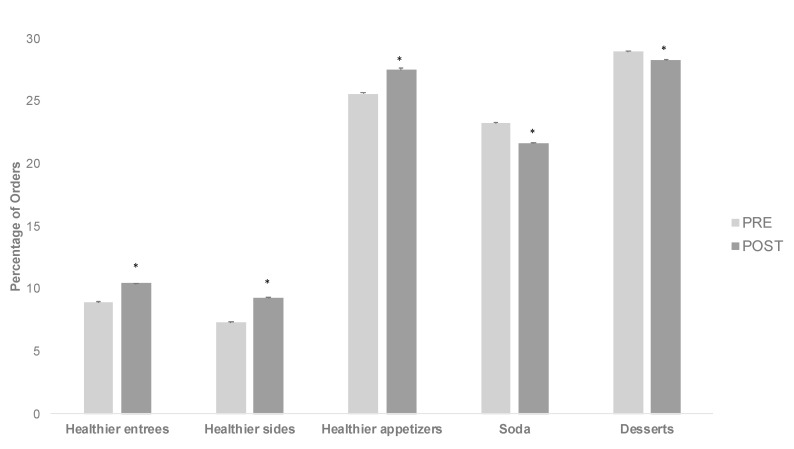
Healthier adult menu orders from before (PRE) to after (POST) the new healthier children’s menu. The healthier children’s menu was implemented in April 2012 at standard locations of a regional restaurant chains. The PRE period (before the healthier children’s menu was implemented) was September 2011 to March 2012. The POST period (after the healthier children’s menu was implemented) was September 2012 to March 2013. Items classified as healthier were designated as healthier options on the adult menu. Orders of soda and entrées that included desserts were also evaluated. * denotes that PRE to POST differences in the percentage of orders that included healthier items, soda, or dessert were statistically significant based on McNemar tests of paired proportions; asymptomatic *p*-values for all tests <0.0001. Standard errors are also shown.

Offerings designated as healthier options were not prominent features of the adult menu and were consistently offered across time (Table 2). The percentage of orders that included healthier entrée, side, and appetizer options increased from PRE to POST from 8.9%(0.02) to 10.4%(0.02), 7.3%(0.02) to 9.3%(0.02), and 25.5%(0.15) to 27.5%(0.17), respectively (Figure 1). Orders of soda and dessert decreased during that same time from 23.2% (0.04) to 21.7% (0.04) and 29.0% (0.03) to 28.3% (0.03), respectively (Figure 1). All PRE to POST changes were statistically significant (*p* < 0.0001; Figure 1).

The majority of meal orders at both time points included breakfast items, which could be ordered all day (Table 3). Overall, the same top five entrée, side, appetizer, and beverage items were the most popular across both time points (Table 3). However, the percentage of orders that included the most popular of the healthier entrée, appetizer, and side and non-soda beverage items increased significantly between PRE and POST (Table 3; *p* < 0.0001).

## 4. Discussion

Our findings demonstrate small but significant increases in the orders of healthier adult menu options during the same time period as the healthy children’s menu change. The percentage of healthier adult menu orders increased after the implementation of the healthier children’s menu and with few changes to what was offered on the adult menu. Over the same time frame, previous research demonstrated larger significant increases in children’s orders of healthy items [5]. We offer several possible explanations for these parallel results and consider their implications in the context of frequent consumption of food from restaurants and evidence of associations with poorer dietary quality and chronic disease risk in adults [2,18,19,20].

First, given the repeated cross-sectional study design, it is possible that we observed improvements in the healthfulness of adult menu orders due to temporal shifts in (a) preferences for healthier options or (b) shifts towards a more health-focused clientele. Existing research suggests that the majority of individuals consider taste rather than health when choosing food away from home [16,27]. Moreover, the nutrition quality of meals eaten at full-service restaurants did not change during this same time frame [28]. Yet there has also been a rise in consumers who identify as health-conscious and new menu offerings often cater to those customers [29,30,31,32,33]. At the top selling restaurant, chains new items have included less calories and saturated fat on average over the study period [32]. At the same time, energy intake from soda and other sugar-sweetened beverages has decreased in recent years [34,35]. Future evaluations of menu change interventions should examine whether there are changes in the consumers’ motivations to patronize the restaurant, whether more consumers cite health as a priority for their choice in where to eat out, and whether parents, in particular, seek out restaurants that have both healthier options for themselves and their children [36,37].

It is also possible that the improvements in the healthfulness of adult menu orders is the result of a spillover effect from the healthier children’s menu on adults dining with children. Spillover may occur as a result of priming from the children’s menu [38] or their desire to serve as a positive role model in the restaurant setting [12]. Spillover effects can serve as a positive feedback loop where children’s orders are improving as a result of the healthier menu, and parents are further serving as a positive influence for their children [13,14,15,16,17,39]. However, the majority of orders placed here were likely by adults not dining with children (adult entrées comprised 18.1% vs. 1.8% of orders for children’s entrées). Furthermore, it is unclear how salient the children’s menu would be for adults dining with or without children. Future research evaluating healthy menu change interventions should asses changes in parents’ motivations for choosing their own meals when dining out and feedback between parents and children in the restaurant setting. Multi-armed experimental studies with parents that test whether parents’ choices change when being exposed to healthier versus less healthy children’s menus could help uncover priming effects from the children’s menu.

While we observed increases in orders of healthier items, the most popular items (such as burgers and pancakes) remained popular at both time points. The consistency in popular items observed here may reflect the habitual patterns of ordering behavior seen in other research in the restaurant setting [16,27,40,41,42]. Habit was cited as one of the most salient factors for ordering decisions at carryout restaurants among low-income African Americans in Baltimore [43] and as a barrier to using calorie labels on menus [44], suggesting that ordering behaviors are difficult to change [45]. As such, the extant literature and present results support strategies that make healthy choices easy and automatic, such as: further promoting already popular healthier menu items and increasing the extent to which healthier items are served by default [5,6,28,46,47,48]. Previous literature suggests that promoting healthier options and offering healthier options by default are effective at increasing orders of healthier items in both children and adults by increasing the automaticity of ordering these healthier items [9,10,46,48,49,50,51,52,53]. Increasing the availability of healthier options on menus also makes these items more normative for both children and parents [53,54].

There were several notable limitations to this study. While these order data from PRE and POST time periods in a single restaurant chain allow exploration of changes in orders of healthier adult menu items, there are several research questions that cannot be addressed. Because these data were aggregated in the point of sale system, we are also unable to determine the nutrition quality of combinations of items ordered by individuals (e.g., entrée, side, and beverage). For example, individuals may have selected a healthier side dish but compensated by ordering a dessert or less healthy entrée. Individual receipt level data were not available for adult orders during this timeframe. It is also possible that individuals rewarded themselves for making healthier choices in the restaurant by eating unhealthy options during other parts of the day. We were unable to detect compensatory behaviors within individuals here. Moreover, we do not know whether the adult orders included in the sales data were from adults dining with children. Future research should evaluate co-occurring responses to healthy menu changes in both parents and children, accounting for potential confounders; the impacts of healthy menu changes on actual consumption (data on the amount of the items ordered that were consumed were not available here); and the potential mechanisms underlying responses to healthy menu changes. Future work employing mixed methods would be especially useful to uncover motivations for adult menu responses and feedbacks between parents and children in the restaurant setting. Given the small changes in adult orders observed here, research uncovering potential mechanisms would also allow for the incorporation of those factors into a multi-pronged intervention that combines successful menu change strategies (i.e., healthy defaults) and targets underlying motivations for healthier orders.

## 5. Conclusions

It is important to consider strategies to improve the healthfulness of adult orders in the restaurant setting, especially given the role of parents in shaping healthy dietary behaviors in children. Our findings demonstrate small increases in the healthfulness of adult menu orders in a restaurant that had implemented healthy changes on its children’s menu. Future research can help elucidate mechanisms underlying improvements to the healthfulness of meal orders and uncover potential dyadic effects between adults and children that impact the overall composition of restaurant meals.

## Figures and Tables

**Table 1:** Calorie and nutrient content of entrées listed as healthier options on the adult menu at a regional restaurant chain.

Nutrient	Mean ± SD	% of Entrées Meeting Cut Point
Calories	503.60 ± 127.4	100
Fat (g)	17.9 ± 8.5	80.0
Cholesterol (mg)	50.0 ± 40.2	92.9
Saturated Fat (g)	3.1 ± 2.3	92.9
Fiber (g)	7.5 ± 3.6	33.3

Notes: These values mean ± standard deviation (SD) were calculated for all entrées listed as healthier options offered at PRE and/or POST. All entrées listed as healthier options were denoted with a heart symbol on the menu. Energy content and nutrition data (as listed on the menus) were used to identify whether entrées listed as healthier options met cut points for calorie, saturated fat, total fat, and fiber needs/limits for a single meal using one-third of the average daily needs/limits for sedentary adults ages 18 and over: 664.3 calories, 7.4 grams of saturated fat, 22.1 grams of fat, and 9.3 grams of fiber, respectively, based on the recommendations in the 2010 Dietary Guidelines for Americans [22].

**Table 2:** Menu items offered on the adult menu before (PRE) and after (POST) the implementation of a healthier children’s menu by meal component.

Item	PRE	POST
**Entrées available on the adult menu**		
Total entrées offered (*n*)	91	89
Entrées offered that were designated as healthier (%)	12.1	12.4
**Sides available on the adult menu**		
Total sides offered (*n*)	30	30
Sides offered that were designated as healthier (%)	16.7	16.7
Appetizers available on the adult menu		
**Total appetizers offered (*n*)**	8	7
Appetizers offered that were designated as healthier (%)	25.0	28.6
**Entrées, sides, and appetizers offered that were designated as healthier options (%)**	14.0	14.3
**Beverages available on the adult menu**		
Total beverages offered (*n*)	13	14
**Desserts available on the adult menu**		
Total adult dessert orders (*n*)	26	27

Notes: Items designated as healthier options were identified via a heart symbol on the adult menu. The PRE period (before the healthier children’s menu was implemented) was September 2011 to March 2012. The POST period (after the healthier children’s menu was implemented) was September 2012 to March 2013. The healthier children’s menu was implemented in April 2012 at standard locations of a regional restaurant chain.

**Table 3:** Adult menu items ordered before (PRE) and after (POST) the implementation of a healthier children’s menu.

Item	PRE	POST
**Food items sold (children’s and adults)**	9,515,446	9,634,257
**Adult entrée orders**		
Total adult entrée orders	1,801,647	1,793,582
Total healthier adult entrée orders	160,948	187,229
Breakfast adult entrée orders (%)	50.5	52.3
*Most ordered adult entrée items (overall)*		
Build your own burger (%)	7.0	6.4
Buttermilk pancake and eggs (%)	4.6	4.9
The American favorite (%)	4.5	4.6
Belgian waffle and eggs (%)	3.0	2.9
Caramel French toast and eggs (%)	2.4	2.5
Most ordered healthier adult entrée items *		
California omelet (%) ^a^	1.5	1.3
Power breakfast (%) ^a^	-	1.3
Baja fish tacos (%) ^a^	1.2	1.2
Low fat vegetarian omelet (%) ^a^	1.1	1.1
600 calorie grilled salmon (%) ^a^	1.1	1.0
600 calorie mango vegetarian stir fry (%) ^a^	0.9	-
**Adult side orders**		
Total adult side orders	2,119,791	2,292,409
Total healthier adult side orders	154,984	213,437
*Most ordered adult side items (overall)*		
Eggs (%)	15.5	19.7
Bacon (%)	11.2	12.1
Toast (%)	8.2	7.5
Sausage (%)	6.8	7.1
Burger (%)	5.0	5.3
French Fries (%)	4.6	6.2
Most ordered healthier adult side items *		
Strawberries (%) ^a^	1.9	5.5
Summer citrus salad (%) ^a^	0.9	0.9
Cholesterol egg free substitutes or egg whites (%) ^a^	0.2	1.7
SD veggie chili (%) ^a^	0.3	0.3
**Adult appetizer orders**		
Total adult appetizer orders	83,101	68,889
Total healthier adult appetizer orders	21,229	18,934
Most ordered adult appetizer items (overall) *		
Buffalo wings (%)	16.2	17.9
Cheese fries (%)	16.1	18.4
Chicken tenders (%)	9.6	11.6
Most ordered healthier adult appetizer items *		
Black bean quesadilla (%) ^a^	14.8	15.2
Goat cheese bruschetta (%) ^a^	10.7	12.2
**Adult beverage orders**		
Total adult beverage orders	1,394,021	1,351,398
Most ordered adult beverage items (overall) *		
Coffee (%) ^b^	31.9	33.3
Juice (%) ^b^	23.6	20.3
Soda (%)	20.1	18.5
Tea (%) ^b^	19.8	19.7
Hot chocolate (%) ^b^	1.9	2.1
**Adult dessert orders**		
Total adult dessert orders	521,585	507,531
Percentage of entrée orders accompanied by a dessert	29.0	28.3

Notes: The top five menu items ordered overall and for healthier options in each category are presented above. For appetizers and beverages, the most popular healthier items were also among the most popular items overall. Soda included fountain beverages such as sprite, coke, and fruit punch. The PRE period (before the healthier children’s menu was implemented) was September 2011 to March 2012. The POST period (after the healthier children’s menu was implemented) was September 2012 to March 2013. ^a^ healthier options were identified via a heart symbol for entrées, sides, and appetizers. ^b^ denotes the items were non-soda beverages (were not fountain beverages, fruit drinks, and/or lemonade). We did not evaluate alcoholic beverages. * denotes that PRE to POST differences in the combined percentage of the top entrée, side, appetizer, and beverage items designated as healthier options were statistically significant based on McNemar tests of paired proportions; asymptomatic *p*-values for all tests <0.0001.
